# Gastric inflammatory myofibroblastic tumor in a 10-month-old girl: A case report

**DOI:** 10.1016/j.ijscr.2020.02.062

**Published:** 2020-03-03

**Authors:** Otto Morales Prillwitz, Bladimir Pérez Hurtado, Stephany Olaya Álvarez, Nasly Marcela Arevalo Sánchez, Raúl Ernesto Astudillo Palomino

**Affiliations:** a Fundación Valle del Lili, Departamento de Cirugía Pediátrica, Cra 98 No. 18-49, Cali, 760032, Colombia; b Fundación Valle del Lili, Departamento de Patología, Cra 98 No. 18-49, Cali, 760032, Colombia; c Fundación Valle del Lili, Centro de Investigaciones Clínicas, Cra 98 No. 18-49, Cali, 760032, Colombia

**Keywords:** IMT, Inflammatory Myofibroblastic Tumor, BP, blood pressure, HR, heart rate, SaO2, oxygen saturation, MRI, magnetic resonance imaging, ALK, anaplastic lymphoma kinase, Inflammatory Pseudo Tumor, Infant, Biopsy, Stomach, Case report

## Abstract

**Introduction:**

Inflammatory Myofibroblastic Tumors (IMTs) are rare mesenchymal tumors of unclear etiology and uncertain malignant potential that affect all age groups. IMTs are most often found in the lungs; although they do occur in a variety of other organs. IMTs have been reported in the mesentery, head and neck, omentum, retroperitoneum, limbs, genitourinary tract, and, extremely rarely, in the stomach. There is scant epidemiological data on IMTs, in part, due to the absence of national and international registries.

**Presentation of case:**

This is a case report of a 10-month-old girl who presented with clinical signs of fever and weight loss over a period of four months. The patient was initially diagnosed with a febrile syndrome of unknown origin. However, upon further investigation, a Gastric IMT was found, and the patient required a left hepatectomy and subtotal gastrectomy with Roux-en-Y reconstruction.

**Conclusion:**

In the pediatric population clinical findings are often nonspecific. Based on this case study, we conclude that, in children with palpable masses, IMT should always be considered as a diagnostic option.

## Highlights

• Inflammatory Myofibroblastic Tumor of the stomach is extremely rare.
• It is a benign mesenchymal lesion that has the potential of local invasion into surrounding organs.
• Complete surgical resection is curative.

## 1 Introduction

This case report has been compiled in accordance with the SCARE 2018 Statement guidelines [1]. The informed consent was obtained from the patient’s mother for publication of this case report.

Inflammatory Myofibroblastic Tumors (IMTs) are rare mesenchymal tumors of unclear etiology and uncertain malignant potential, first described in 1905 by Birch-Hirschfield [2,3]. They commonly affect the lungs, however they have also been discovered at other anatomical sites. Scant epidemiological data is available on these tumors, in part, due to the absence of national and international registries [3,4]. IMTs affect patients of all age groups. In 2011, English-language scientific literature reported 23 cases in children, and 50 cases in adults.

The clinical characteristics are highly variable and relate to the site of origin. IMTs are diagnosed by a palpable incidental mass, and by imaging, accompanied by a histological confirmatory diagnosis. Histology is characterized by prominent spindle cells with inflammatory infiltrate that contains plasma cells, lymphocytes, and eosinophils. These types of tumors are predominantly benign, making radical surgery the preferred treatment option; as the presence of metastases in distant organs has only been described in very few cases [[5], [6], [7]].

This case report concerns a 10-month-old girl with clinical signs of fever and weight loss for a period of four months, initially diagnosed as a febrile syndrome of unknown origin, in whom a gastric IMT was found requiring a left hepatectomy and subtotal gastrectomy with Roux-en-Y reconstruction. The patient was discharged from hospital 23 days after surgery without complications, and was asymptomatic two months after the procedure.

## 2 Presentation of case

A 10-month-old girl, resident of the municipality of Santander de Quilichao, was examined at a local health institution. The patient presented with a four-month unquantified fever, profuse diaphoresis; hyporexia, without vomiting; and weight loss, with no morbidity. A full initial medical examination was performed, during which blood pressure (BP) was found to be 91/69 mmHg, with mean of 76.3 mmHg; heart rate (HR) was 144 beats per minute; and respiratory rate was 27 breaths per minute. Oxygen saturation (SaO2) was 94 %, and temperature 36.6 °C. A palpable, non-mobile mass was discovered in the mesogastrium and epigastrium of the abdomen. The mass was not painful, and there were no other relevant observable symptoms. Laboratory tests indicated: white-cell levels of 31,600 cells/mm^3^, neutrophils at 75 % (23.4 × 103 cells/μL), hemoglobin levels of 4.6 g/dL, hypertrophic cardiomyopathy (HCM) of 18 pg/mL, platelets at 1,054,000 cells/mm^3^, and C-reactive protein levels of 27.9 mg/dL. The patient received a blood transfusion comprising 100 ml of red blood cells.

**Fig. 1:**
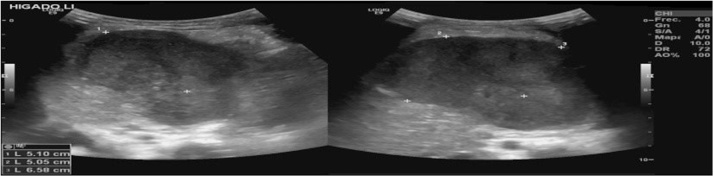
Two ultrasound images of the same tumor. Rounded hypoechoic mass between the left lobe of the liver and the spleen. Approximately 52 × 59 × 56 mm.

A total abdominal ultrasound was performed (Fig. 1) showing a solid abdominal mass of 52 × 59 × 56 mm, with well-defined contours, located between the spleen and the liver with a volume of approximately 120 cm^3^. In order to prevent unfocused sepsis and anemia with hemodynamic repercussions, antibiotic coverage with Cefepime plus Vancomycin was prescribed.

Tests were conducted for the presence of any infectious diseases, including: HIV, VDRL serology, Immunoglobulin M for leptospira, Malaria microscopy, and tuberculin.

All blood cultures, urine cultures and cultures for rectal and nasal screening returned negative results: Anti-Hepatitis B antigen >1000, Toxoplasma IgG 0.47 and IgM 0.4, Cytomegalovirus IgG 3.10 and IgM 0.73 were non-reactive, Epstein Barr IgG 3 and IgM 4.2 were negative, as was the BK sputum series, and the gastric aspirate was negative for acid resistant bacilli. Immunodeficiencies were excluded by examining levels of immunoglobulins: IgG 1153 mg/dL, IgA 193.3 mg/dL, IgM 252.8 mg/dL, IgE 428.2 mg/dL. Hemogram results showed normal levels of: Leukocytes 45,470 cells/mm^3^, Neutrophils 82 % (35.57 × 10^3^ cells/μL), Platelets 868,000 cells/mm^3^, Hemoglobin 7.5 g/dL, VCM 74.7 fl, HCM 21.7 pg/mL, C-reactive protein 34.4 mg/dL, Albumin 2.9 g/dL. The levels of LDH (207.3 mg/dL), Alpha-Fetoprotein (11.08 ng/mL) and BHCG (<0.1 mUI/mL) were unaltered in peripheral blood after extended analysis and coagulation times. Renal and liver functions appeared normal.

**Fig. 2:**
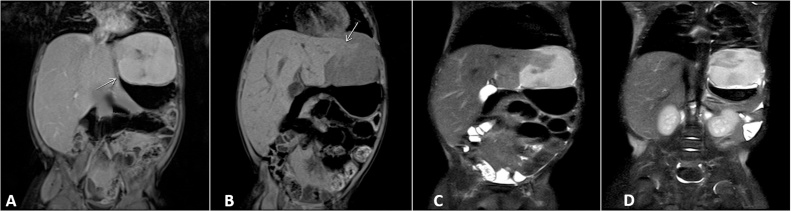
Abdominal RMI showing 5.5 × 5.9 cm mass of lobed and well-defined contours that involves: (A) Gastric body (B) Segment II liver and diaphragm. (C–D) Enhancement is observed in the contour of the mass in T2.

Based on the findings of the abdominal ultrasound, magnetic resonance imaging (MRI) with contrast of the abdomen was requested in order to determine the characteristics and location of the mass (Fig. 2). MRI imaging showed a solid mass with lobulated edges, and well-defined contours of peripheral subcapsular localization on the left lateral margin of the II hepatic segment, with a diameter of 55 × 87 × 59 mm. Initial diagnosis proposed that the mass might be a hemangioma, and focal nodular hyperplasia was put forward as a second diagnostic possibility.

An exploratory laparotomy was carried out to determine the etiology of the abdominal mass. During exploration of the abdominal cavity we found: intestinal malrotation and a tumor of gastric origin of 120 × 70 × 60 mm that involved the left lobe of the liver, left diaphragm, and esophageal lymph nodes. A left hepatectomy, subtotal gastrectomy with Roux-en-Y reconstruction and resection of paraesophageal lymph nodes were performed, during which the whole tumor was resected. All samples were sent for pathological and immunohistochemical testing.

**Fig. 3:**
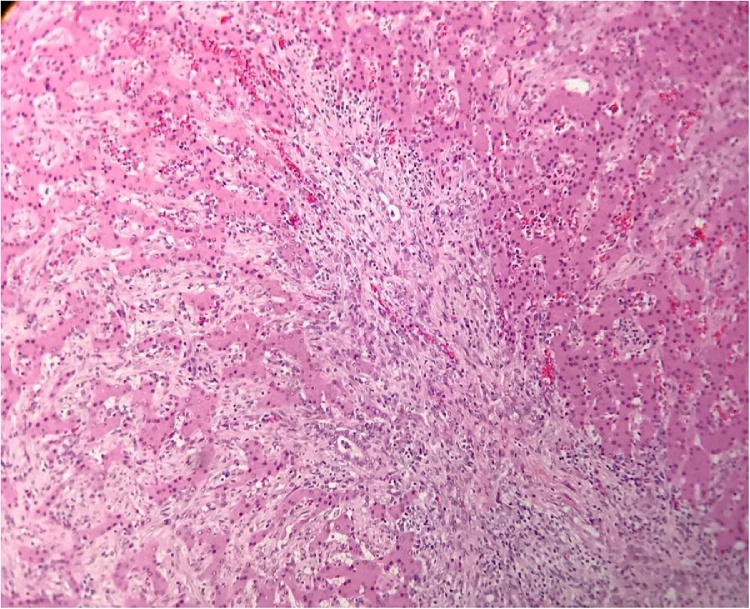
HE 20×. Transition between the hepatic and neoplastic parenchyma. This lesion has an infiltrating pattern in relation to the liver.

**Fig. 4:**
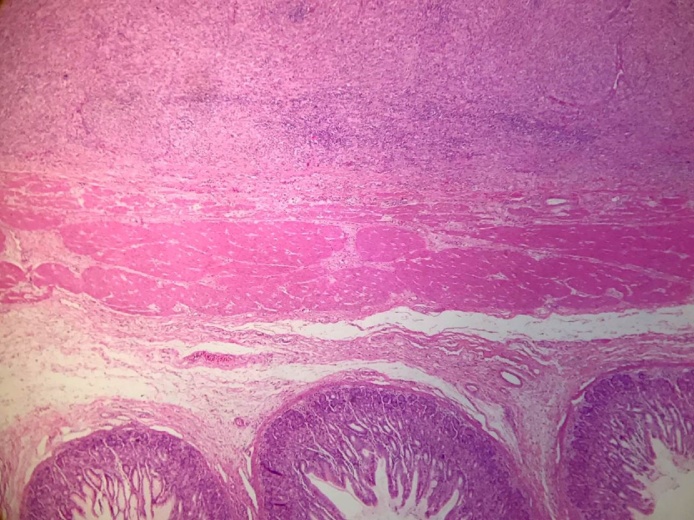
HE-4×. Mucosa of the stomach in transition with the neoplasia. Between the muscle itself and the serous without infiltrating it.

**Fig. 5:**
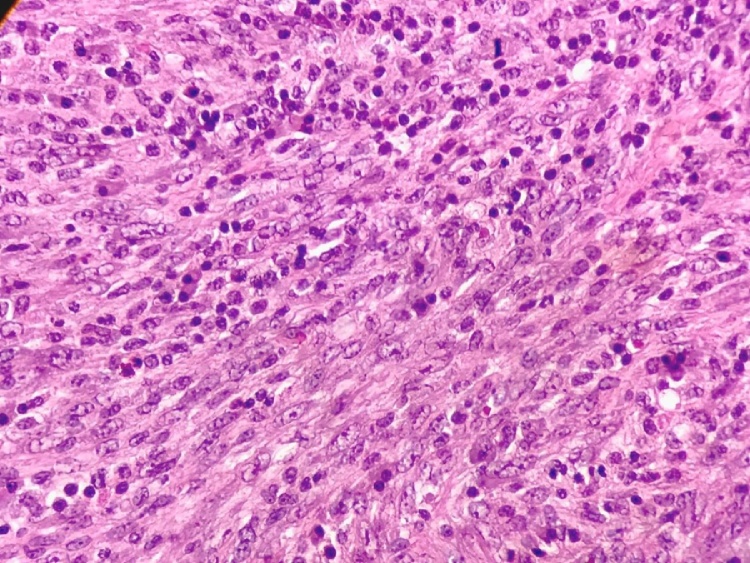
HE 40×. Neoplastic cells mixed with inflammatory cellularity composed of lymphocytes and plasma cells.

Macroscopic examination of the resected specimen revealed a completely encapsulated bright grayish tumor, with a firm consistency, and nodular appearance. Microscopically, neoplastic cellularity expanded into the gastric mucosa and infiltrated the hepatic parenchyma (Fig. 3). The resected tissue consisted of a well-encapsulated mesenchymal neoplastic lesion with spindle cells present in a storiform and fascicular pattern (Fig. 4). The cells forming the neoplasia are long with eosinophils and scarce cytoplasm. The nuclei are regular, with evident nucleoli and no cytologic atypia or mitosis. These cells are interspersed with inflammatory cellularity characterized by a predominance of lymphocytes and plasma cells (Fig. 5). The diaphragmatic, hepatic, and gastric resection edges were free of tumor infiltration.

**Fig. 6:**
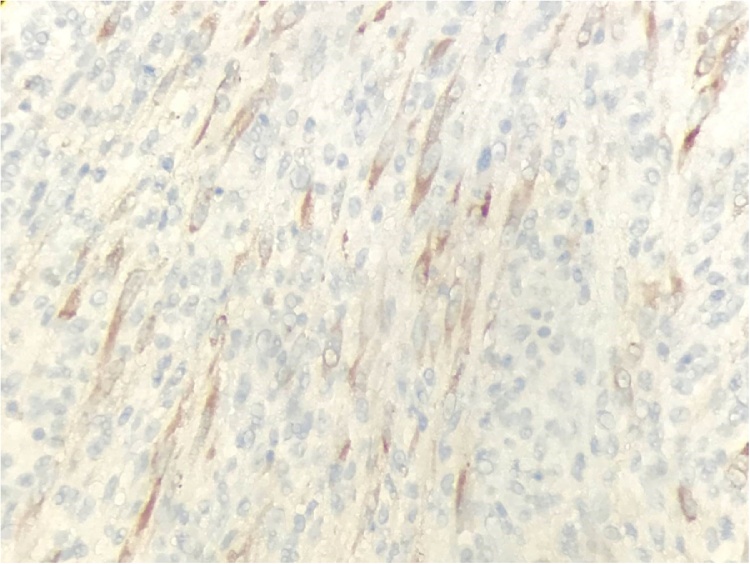
AL 40×. Cytoplasmic positivity in Neoplastic Cells.

In immunohistochemical studies, the cells showed positivity for anaplastic lymphoma kinase (ALK), heterogeneous positivity for BCL2, and heterogeneous and weak positivity for CK AE1-AE3 and Muscle Specific Actin, as well as positivity, in the “Myofibroblastic” pattern, for Calponin and Muscle Specific Actin (Fig. 6). All cells were negative for desmin, S-100, CD117, CD34, IgG, and IgG4. The proliferative index of ki-67 is 18 %. These findings confirm the histological diagnosis of IMT. Histological sections showed six lymph nodes without alterations.

In the postoperative period, the patient showed an improvement in acute phase protein levels. An intolerance to the oral route diminished with the use of prokinetics, and there were no further post-surgical complications.

The patient was discharged 23 days after the surgical procedure in a good condition (i.e., vital signs were stable and within normal limits, she was conscious and comfortable, and indicators for recovery were excellent). She had remained asymptomatic for two months at the time of preparation of this case report. Table 1 summarizes the results of the hemograms conducted before and after the surgical procedure.

**Table 1:** Blood count behavior before and after resection of a gastric myofibroblastic inflammatory tumor.

Date	Entry	Postoperative	Egress
Leukocytes × 10^3/μL	31,6	19,5	8,7
Neutrophils x 10^3/μL	23,4	13,2	2,6
Neutrophils (%)	75	67,8	30,6
Hemoglobin g/dL	4,6	13,5	13,9
Hematocrit %	18	40,8	43,5
Platelets × 10^3/μL	1054	487	258
PCR	27,9	4,6	0,8

## 3 Discussion

IMT are rare, benign mesenchymal lesions with variable location that can occur at any age, with a predominance in pediatric patients [8]. While the exact etiology of IMTs remains unclear, an infectious origin is suspected due to the presence of inflammatory characteristics and the elevation of acute phase reactants. Some of the most frequently associated infectious agents include: *Mycobacterium avium intracellulare*, *Corynebacterium equi*, *Bacillus sphaerius*, *Coxiella burnetii*, *Epstein Barr virus,* and fungi [9]. IMTs may also arise as a secondary response to trauma [9,10].

IMT occurring in the stomach is relatively rare in children, and even less frequent in children under one year old. In Japan, Hayashi et al. reported a case in which a one-month-old girl presented with an ITM located in the pylorus [11]. In Germany, Hirschburger et al. reported a second case in an eight-month-old girl presenting with an ITM located in the minor curvature of the stomach [12]. The present case is the first to be reported in which an IMT was found in the stomach of a child younger than one year old, in the southwest of Colombia.

Gastric IMT usually presents with epigastric pain, hematemesis, melena and a palpable abdominal mass [13]. Microcytic hypochromic anemia may occur over time, secondary to the formation of intragastric polypoid ulcers [14]. The most common constitutional signs and symptoms are fever and weight loss, secondary to the release of inflammatory cytokines, such as Interleukin-6 [15]. Few cases of primary gastric IMT have presented only with fever of unknown origin refractory to antibiotic therapy [15], as was the case with our patient.

There are no conclusive laboratory tests available for the diagnosis of IMT, and imaging alone does not provide specific information. However, given the inflammatory characteristics, the following test results should be used as indicative during the diagnostic protocol: complete blood counts, where leukocytosis with left deviation are often found; thrombocytosis; hypochromic microcytic anemia; elevation of the rate of erythrocyte sedimentation; and increase of C-reactive protein [7]. Although ultrasound imaging identifies the location of lesions in the abdominal and pelvic cavity, and computed tomography and magnetic resonance imaging allow us to determine the tumor size, site, and relation to neighboring structures [7], a definitive diagnosis can only be achieved through histological examination of tissue samples obtained by biopsy. Histology shows the presence, at microscopic level, of chronic inflammatory cells, comprising proliferating spindle cells (myofibroblasts), within a variable stroma, between a myxoid or dense collagen matrix, with elongated vesicular nuclei and inclusions, such as nucleoli [16]. Immunohistochemistry may still lead to multiple differential diagnoses, such as gastrointestinal stromal tumors (GISTs), fibromatosis, retroperitoneal fibrosis, leiomyosarcoma, and malignant peripheral nerve injury [3], however IMT shows immunohistochemical positivity for vimentin, smooth muscle actin, and desmin. It has been reported that 56 % of IMT cases are positive for the presence of ALK. This result has been related to clonal abnormalities, involving the ALK gene that is located on chromosome 2p23, which appears to support the neoplastic nature of IMTs. There is an open discussion about the importance and impact of ALK-score positivity on IMT prognosis. studies have reported less aggressive tumor behavior in patients with positive ALK scores, although these results have been just as varied [3].

ALK immunohistochemistry is a useful diagnostic supplement in appropriate circumstances, when ALK is positive and other markers such as CD34, CD21, CD117, CD23, and S100 are negative, these results support the clear pathological diagnosis of IMT and exclude all other possibilities, such as GISTs, or inflammatory fibroid polyps [17,18].

The preferred treatment method for IMT is complete surgical resection. However, there are cases in which the tumor has invaded vital structures in the thoracic or abdominal locations, and, in these cases partial resection is recommended, particularly given the benign nature of IMTs [[2], [3], [4],^6^]. Clinical follow-up should be continued and the patient monitored very closely, with regular imaging studies to check for signs of recurrence.

The recurrence of IMTs, according to previous studies, varies widely between cohorts. Recurrence frequencies as low as 3% have been reported in some patient cohorts, whereas they can be as high as an average of 44 % in others [3]. Recurrence may occur at any time from three months post-surgery up to seven years after surgical resection. When recurrence occurs, the preferred treatment course is surgical resection, although some studies recommend adjuvant chemotherapy as a further treatment option, citing positive results in terms of recurrence and/or metastasis [17].

The presence of malignancy from IMT is very rare, and such cases are often characterized by a marked clinical deterioration and aggressive tumor behavior with recurrence and/or metastases. Chemotherapy and radiotherapy have been shown to be the most effective treatment regimens for malignant IMTs [6].

## 4 Conclusion

The presentation of cases with IMT is variable, with the most common symptoms being fever, elevated levels of acute phase reactants, and the presence of a palpable mass. Diagnostic images allow us to identify tumor location and guide the surgical procedure. Immunohistochemistry, along with pathology, are the most reliable diagnostic methods for the identification of gastric IMTs.

IMTs usually exhibit benign characteristics, leading to favorable prognoses. In pediatric patients, presenting with nonspecific clinical findings and palpable masses, an IMT should always be considered as a diagnostic option.

## Funding

This research did not receive any specific grant from funding agencies in the public, commercial, or not-for-profit sectors.

## Ethical approval

This case report was approved by the IRB/EC of our institute, Reference number 243-2017.

## Consent

Written informed consent was obtained from the Mother of patient for publication of this case report and accompanying images. A copy of the written consent is available for review by the Editor-in-Chief of this journal on request.

## Author contribution

Dr. Otto Morales Prillwitz contributed to: Study concept, analysis and interpretation. Also in writing the paper.

Dr. Bladimir Perez contributed to: Study concept, analysis and interpretation and writing the paper.

Dra. Stephany Olaya contributed to: Data collection, analysis, interpretation and writing the paper.

Dra. Marcela Arevalo contributed to: Data collection, analysis, interpretation and writing the paper.

Dr. Raul Ernesto Astudillo contributed to: Study concept, analysis and interpretation.

## Registration of research studies

None.

## Guarantor

Dr. Otto Morales Prillwitz.

## Provenance and peer review

Not commissioned, externally peer-reviewed.

## Declaration of Competing Interest

The authors declare no conflict of interest.
